# An improved method for detecting and delineating genomic regions with altered gene expression in cancer

**DOI:** 10.1186/gb-2008-9-1-r13

**Published:** 2008-01-21

**Authors:** Björn Nilsson, Mikael Johansson, Anders Heyden, Sven Nelander, Thoas Fioretos

**Affiliations:** 1 Department of Clinical Genetics, Lund University Hospital, SE-221 85 Lund, Sweden; 2 Department of Transfusion Medicine, Lund University Hospital, SE-221 85 Lund, Sweden; 3 Imaging Platform, Broad Institute of Harvard University and MIT, Cambridge, MA 02142, USA; 4 Department of Automatic Control, Royal Institute of Technology, SE-100 44 Stockholm, Sweden; 5 Department of Applied Mathematics, Malmö University, Malmö, SE-205 06 Malmö, Sweden; 6 Computational Biology Center, Memorial Sloan-Kettering Cancer Center, New York, NY 10021, USA

## Abstract

Genomic regions with altered gene expression are a characteristic feature of cancer cells. We present a novel method for identifying such regions in gene expression maps. This method is based on total variation minimization, a classical signal restoration technique. In systematic evaluations, we show that our method combines top-notch detection performance with an ability to delineate relevant regions without excessive over-segmentation, making it a significant advance over existing methods. Software (Rendersome) is provided.

## Abstract 2

A method is presented for identifying genomic regions with altered gene expression in gene expression maps.

## Background

Alterations in gene expression patterns, resulting from acquired genetic and epigenetic changes, are a characteristic feature of cancer cells. Recently, several studies have shown that the expression of a considerable fraction of genes located in regions of gains or losses of chromosomal material varies consistently with DNA copy number, leading to altered (biased) gene expression in such regions [1-11]. Conversely, additional studies suggest that gene expression biases inferred from expression maps are either caused by underlying genomic imbalances [12-17] or long-range epigenetic mechanisms, including DNA methylation or histone modification across large chromosomal regions [18,19]. Thus, the analysis of microarray data from tumors with respect to alterations in regional gene expression is potentially useful for studying relationships between DNA copy number and gene expression, mining pre-existing expression array data for imbalanced chromosomal aberrations [20] or identifying genomic regions that are susceptible to epigenetic change [19].

A central problem associated with the identification of genomic regions with biased gene expression is to partition the expression map into contiguous regions that share the same baseline expression level (bias) on average. This process, called *segmentation*, serves to reconstruct (or restore or de-noise) the underlying expression bias profile from the primary data, and to detect relevant regions and delineate their boundaries. In principle, segmentation of expression maps is analogous to reconstructing DNA copy number profiles from array comparative genome hybridization (aCGH) or single nucleotide polymorphism (SNP) arrays. However, additional challenges are present that make the problem harder. First, the genomic resolution of expression arrays is coarser, that is there are fewer probes per chromosome. Second, the signal-to-noise ratio (SNR) is lower, in the sense that the expression biases we aim to detect are moderate in comparison with the intrinsic variability in gene expression. Third, the expression of some genes may not be influenced by the underlying genomic change. For example, copy number gains are unlikely to increase the expression of genes whose necessary transcriptional activators are absent.

In the present study, we describe an improved method for detecting and delineating genomic regions with biased gene expression in cancer. The proposed method differs from previous proposals in two important respects. First, the method is based on total variation (TV) minimization, a classical approach for recovering signals or images corrupted by noise [21]. Second, whereas existing segmentation methods target aCGH and SNP data, our method is optimized for expression microarray data. We show how to adapt the TV minimization technique for the segmentation of gene expression maps and derive efficient algorithms for its computation. In systematic evaluations, we show that segmentation by TV minimization combines enhanced detection performance with an enhanced ability to delineate relevant regions, making it a significant advance over existing segmentation techniques. We also verify that our method is capable of identifying regions with expected increases/decreases in the average level of gene expression, in this case on the basis of known imbalanced chromosomal aberrations in childhood acute lymphoblastic leukemia (ALL). Finally, we provide a software package, Rendersome, which is publicly available.

## Results

### Evaluation by simulation

**Figure 1:**
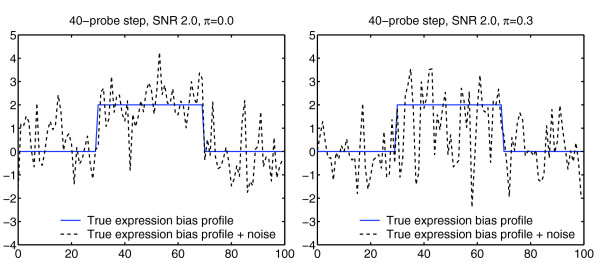
**Simulation model**. Blue solid: Original gene expression bias profile containing a centrally located region with increased expression. Black dotted: Corresponding gene scores, generated by mixing a high-frequency signal component into the original bias profile (details in Materials and methods). Left: Example signal generated with 40-probe step with SNR 2.0, and no non-influenced genes (*π *= 0.0). Right: Corresponding signal with a higher proportion of non-influenced genes (*π *= 0.3).

We first performed a series of simulations, which were designed to assess the ability of the proposed method to identify genomic regions with biased gene expression under varying conditions. As described in detail in Materials and methods, we repeatedly simulated artificial 'chromosomes' containing a centrally located biased region (a square wave step), mixed with a randomly generated high-frequency signal corresponding to noise plus the intrinsic variability in expression between genes (Figure 1). The type of expression profiles generated by this model is controlled by four parameters: the length of the chromosome, the width of the biased region in the center, the SNR and the proportion of genes (*π*) that are not influenced by the underlying genomic alteration. By varying these parameters, we could artificially recreate gene expression maps with a wide variety of signal characteristics.

To ensure comprehensive testing, we selected parameter combinations from broad and relevant intervals (Materials and methods). For each set of parameters, we generated a series of artificial chromosomes and assessed the detection performance, delineation performance and the visual performance of the proposed method plus a control method. As control methods, we considered CGHseg by Picard et al [22] and DNAcopy by Olshen et al [23]. These methods have been evaluated recently by extensive simulation and by application to real data [24,25] and were found to compare favorably to other segmentation techniques. In particular, Lai et al [24] noted that CGHseg, followed by DNAcopy, performed consistently well for a broad range of conditions, including low SNRs which is the most relevant case here. In agreement with [24], we found CGHseg to perform better than, or on par with, DNAcopy (data not shown). Hence, we selected a CGHseg as a state-of-the-art method to compare our results with.

### Detection performance

We first computed the receiver operating characteristics (ROC) curves for each segmentation technique to assess the detection performance (that is, the trade-off between sensitivity and specificity for detecting relevant regions) in each case. To generate ROC curves for specific combinations of simulation parameters, we calculated the true positive rates (TPRs) and false positive rates (FPRs) across 200 simulated 100-probe chromosomes as we varied the threshold for calling probes relevant (Materials and methods). This approach has been previously established as an appropriate way to evaluate segmentation methods [24,25].

**Figure 2:**
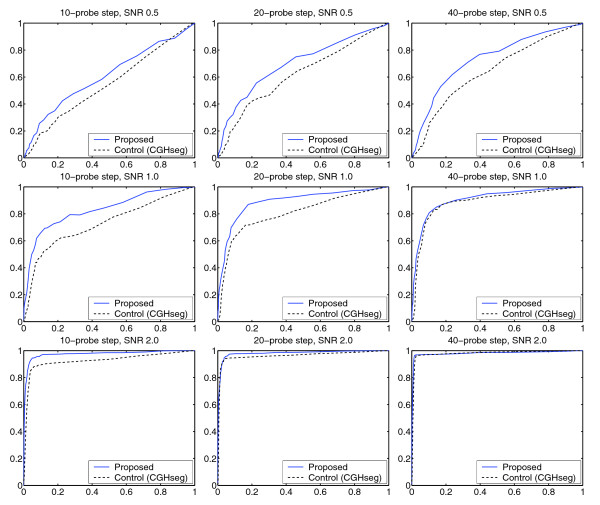
**Receiver operating characteristics**. To assess the ability of the proposed method to detect genomic regions with biased gene expression, we determined its ROC curve for different SNRs, aberration sizes and proportions of non-influenced genes (Materials and methods and also Figure 1). This figure (*π *= 0.1) represents an excerpt from the full set of results (Additional file 1). Key observations: (1) the proposed method exhibits stronger detection performance than the control method (CGHseg); (2) the improvement is present throughout, but is particularly pronounced for low to intermediate SNRs. We conclude that the proposed method exhibits a better trade-off between sensitivity and specificity, especially under expression data-like conditions.

As shown in Figure 2 and Additional file 1, the proposed method exhibited considerably stronger ROC curves. The difference was present throughout, and was particularly pronounced for low to intermediate SNRs (the most expression data-like conditions). The proposed method also displayed the best performance when the proportion of 'non-influenced genes' was high. We conclude that the proposed algorithm offers an improved trade-off between sensitivity and specificity when determining aberration, especially under conditions that are likely to apply in real gene expression maps.

### Delineation performance

We next assessed the ability to delineate the boundaries of relevant regions. To achieve this, we generated and segmented 10,000 artificial chromosomes for each set of simulation parameters. Based on the segmentation results across all chromosomes, we computed the relative breakpoint frequency at each chromosomal position. In doing this, we obtain a set of 'breakpoint maps' that reveal how often, and how precisely, a segmentation method identifies the true breakpoints (Materials and methods).

**Figure 3:**
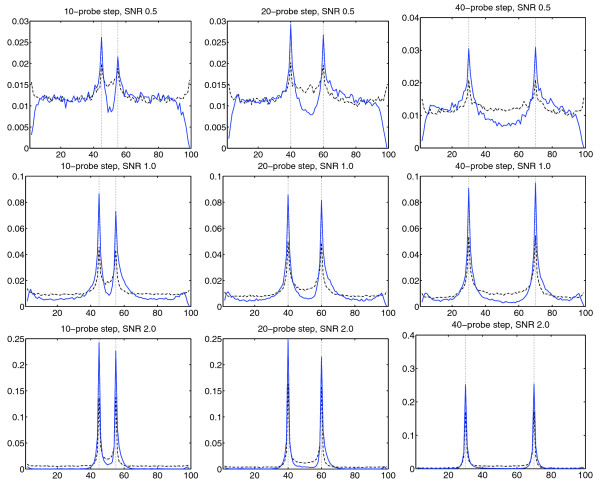
**Breakpoint distributions**. To assess the ability of the proposed method to delineate relevant regions, determined its breakpoint distributions for different simulation parameters (Materials and methods). This figure (*π *= 0.1) represents an excerpt from the full set of results (Additional file 2). Key observations are as follows. (1) The distributions of the proposed method exhibit significantly higher 'peaks' around the true breakpoints (vertical dotted lines). This signifies that, given that the proposed method detects a breakpoint, the probability that it is a true breakpoint is higher. (2) The distributions for the proposed methods exhibit markedly 'scooped' centers, that is, there is less distributional mass (fewer breakpoints) inside the relevant segment. Thus, the method detects fewer false breakpoints inside relevant regions, even when the region is large. This improvement is a result of the use of multiple regularization parameter values (Materials and methods). (3) As in Figure 2, the improvements were particularly pronounced under expression data-like conditions. In this test, *T*_*μ *_= 0.5·SNR (similar results for other reasonable values).

As shown in Figure 3 and Additional file 2, the breakpoint distributions of the TV-based segmentation scheme stand out in two important respects. First, the proposed algorithm yielded higher histogram peaks at, or near, the true breakpoints (in our case, the edges of the centrally located biased region). Thus, given that the algorithm reports a breakpoint, the probability that it is located at, or near, a true breakpoint is higher. Second, the breakpoint distributions of the proposed algorithm display a markedly 'scooped' center, that is there is little distributional mass (fewer breakpoints) inside the relevant region.

Interestingly, this finding signifies that the TV-based scheme, to a great extent, manages to avoid reporting false breakpoints inside relevant regions. This improvement is a result of the fact that the proposed method explicitly seeks to segment relevant regions 'in one piece' (Materials and methods). The differences in breakpoint distribution could be observed throughout but were particularly pronounced for low and intermediate SNRs (Additional file 2). We conclude that, in addition to stronger ROC curves, the proposed algorithm identifies the correct region breakpoints with higher probability and detects relevant regions without excessive over-segmentation.

### Visual performance

**Figure 4:**
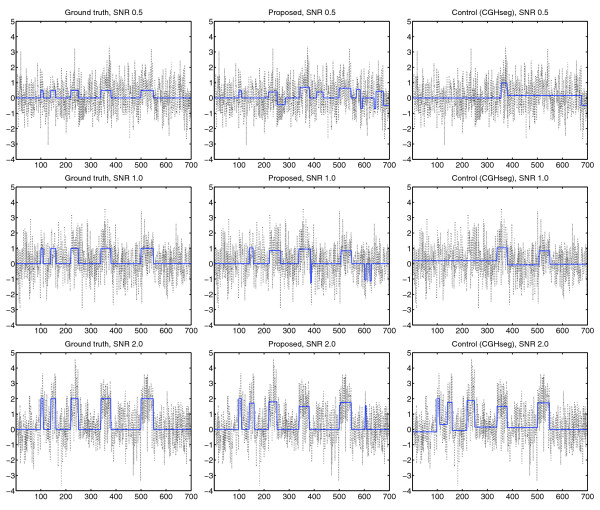
**Application to synthetic data**. For illustration, we applied the different methods to a large set of synthetic examples. Left: Original gene expression bias profile. Middle: Results for proposed method. Right: Results for CGHseg. As evident, the proposed method better succeeds in recovering the true expression bias profile, especially under rough conditions. The example shown was generated using *π *= 0.2, but agreeing results were obtained for *π *= 0.0 to 0.5. In this test, *T*_*μ *_= 0.5·SNR (similar results for other reasonable values).

As the third and final part of the performance comparison, we decided to examine segmentation results obtained on simulated examples. As before, we mixed a piece-wise constant expression bias profile into a randomly generated high-frequency component (as described in the Simulation model section). In this case, however, we placed five biased regions of varying widths (10, 20, 30, 40 and 50 probes) along the same chromosome (Figure 4). For each combination of SNR and proportion of non-influenced genes, we generated and inspected 10 examples visually. Throughout, the TV-based scheme generally produced segmentation results that more closely resembled the original (uncorrupted) signal. Admittedly, visual evaluations of this type are prone to subjectivity and should be interpreted with caution. Still, the results obtained were consistent with, and partially explain, the improvements observed in the first two experiments.

### Application to real data

We proceeded to apply TV minimization-based segmentation to real expression microarray data to verify its ability to identify regions with expected increases/decreases in average gene expression. To achieve this, we used the data set generated by Ross et al [26], consisting of expression profiles of childhood ALLs, classified by genetic subtype (Table 1). This disease subclassification builds on cytogenetic and molecular genetic criteria, and is instrumental for the diagnostic, prognostic and therapeutic stratification of ALL patients in clinical practice [27]. Of interest here is that each genetic subtype is characterized by recurrent, well-defined chromosomal aberrations [28]. Some of these aberrations are balanced translocations whereas some are imbalanced aberrations (gains or losses of chromosomal material). The latter type of aberration alter the DNA copy number (the 'gene dose') and hence can be expected to cause increased/decreased gene expression across the engaged chromosome or chromosomal segment. We seek to test whether the proposed method succeeds in identifying regions that correspond to common imbalanced chromosomal aberrations in specific leukemic subtypes.

**Table 1:** Characteristics of the test data. Contents of the Ross data set [26] of expression profiles of childhood acute lymphoblastic leukemias (ALL). The elements indicate the numbers of cases of each leukemic subtype, as defined by cytogenetic and molecular genetic criteria according to the World Health Organization (WHO) classification system [27]. Also outlined are the clinical characteristics and defining genetic change of each leukemic subtype.

Leukemic subtype	Number of cases	Clinical characteristics
B-cell ALL, Hyperdiploid (> 50 chromosomes)	17	Around 25% of childhood ALL cases, favor-able prognosis, gains of chromosomes X, 4, 6, 8, 10, 14, 17, 18 or 21.
B-cell ALL, *TCF3/PBX1 *gene fusion	18	Around 5% of cases, poor prognosis without intensive treatment, gene fusion corresponds to a balanced translocation between chromo- somes 1 and 19.
B-cell ALL, *ETV6/RUNX1 *gene fusion	20	Around 25% of cases, favorable prognosis, gene fusion corresponds to a balanced trans- location between chromosomes 12 and 21.
B-cell ALL, *BCR/ABL1 *gene fusion	15	Around 3% of cases, unfavorable prognosis, gene fusion corresponds to a balanced trans- location between chromosomes 9 and 22.
B-cell ALL, *MLL *fusions	20	Around 80% of cases in infants, about 5% of older children, unfavorable prognosis, gene fusions correspond to various structural re- arrangements of chromosome band 11q23.
T-cell ALL	14	Unfavorable prognosis.

The technical details are given in Materials and methods. In short, all expression data were converted to a log-scale, normalized with respect to out-of-class cases and then segmented. The original and segmented data were plotted, both case-by-case and class-by-class. The class-by-class plots represent the average segmentation result across all cases of each leukemic subtype, and hence emphasize recurrent alterations in expression while suppressing sporadic changes and noise. To provide a map of frequent imbalanced chromosomal aberrations in ALL we overlaid average DNA copy number profiles for each leukemic subtype, as computed from high-resolution SNP array data by Mullighan et al [29]. The copy number profiles indicate which regions that can be expected to show increased/decreases in expression on the basis of common gains or losses of chromosomal material, but do not indicate regional biases that have other causes.

**Figure 5:**
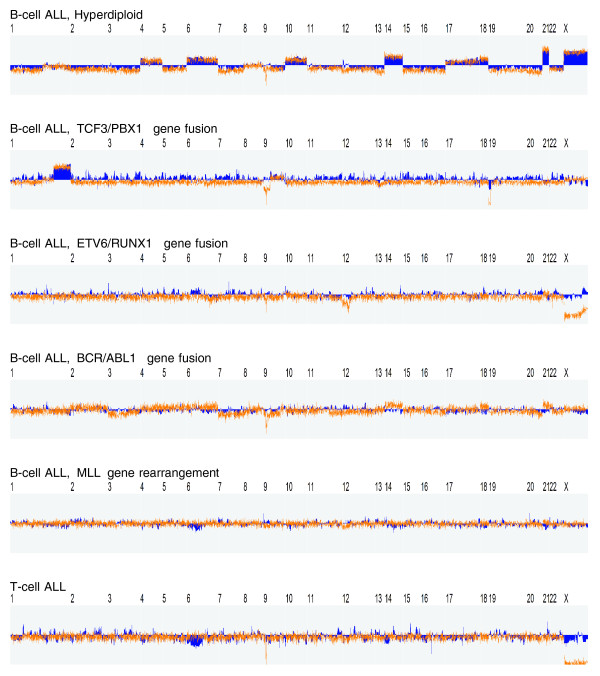
**Application to childhood ALL data**. To verify the ability of the proposed method to identify genomic regions with expected increases/decreases in average gene expression, we applied it to the data set by Ross et al [26] (Affymetrix U133A+B arrays). Each case was normalized and segmented as described in Materials and methods. Blue solid: Average segmentation result across all cases of each leukemic subtype (Table 1). Orange: Average DNA copy number profile across within each class, as determined from the Mullighan et al data set [29] (Affymetrix 250 k SNP arrays). Key observation: The method successfully identified several regions with altered gene expression (details in Results). The case-specific segmentations are provided in Additional file 3. In this example, *T*_*μ *_= 0.25 (similar results for other reasonable values).

**Figure 6:**
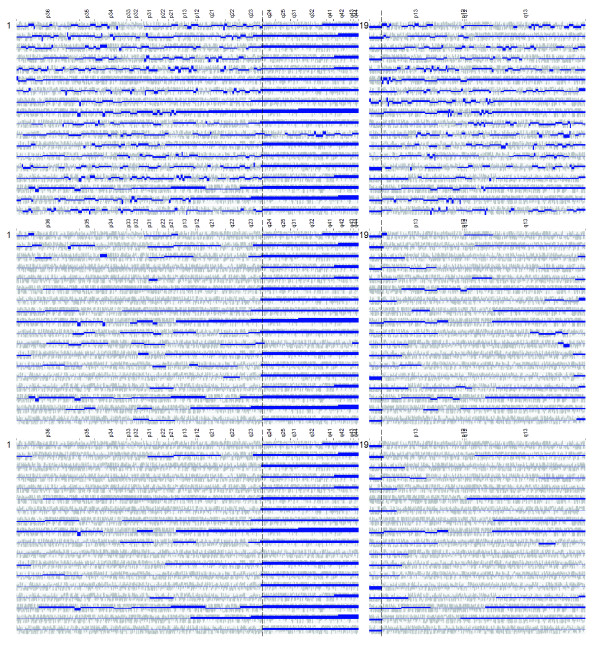
**Application to childhood ALL with TCF3/PBX1 gene fusion**. Segmentations of the expression maps of chromosomes 1 and 19 in 18 cases of ALL exhibiting the *TCF3/PBX1 *fusion oncogene (Ross et al data set) using different method parameters. Light grey: original gene scores. Dark blue: reconstructed expression bias profile. Top: *λ*_*N *_= 2/5. Middle: *λ*_*N *_= 2/15 Bottom: *λ*_*N *_= 2/30. Key observations are as follows. (1) Most cases display over-expression in 1q distal to the *PBX1 *locus and under-expression over a ~1.6 Mb region on 19p distal to the *TCF3 *locus (translocation breakpoints indicated by vertical bars). The explanation for this finding is discussed in the Results section. (2) Reducing *λ*_*N *_allows the algorithm to emphasize on larger regions, while suppressing smaller regions.

**Figure 7:**
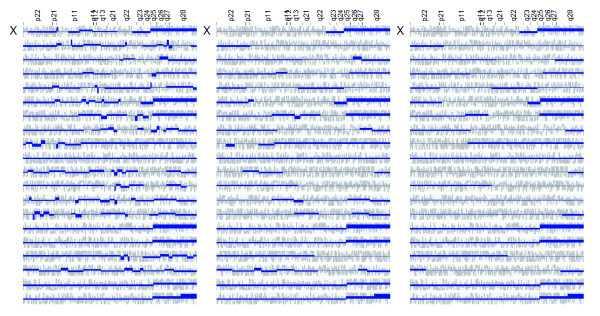
**Application to childhood ALL with ETV6/RUNX1 gene fusion**. Segmentations of the expression map of the X chromosome in 20 cases of ALL harboring the *ETV6/RUNX1 *fusion. Light grey: original gene scores. Dark blue: reconstructed expression bias profile. Top: *λ*_*N *_= 2/5. Middle: *λ*_*N *_= 2/15 Bottom: *λ*_*N *_= 2/30. Key observations are as follows. (1) Several cases exhibited over-expression in Xq25-28, a chromosomal region that was not known to be recurrently gained in *ETV6/RUNX1*-positive ALL until recently. Following more detailed investigations at our lab using aCGH, the region was shown to be frequently duplicated in this leukemic subtype [20]. Thus, this finding further supports that the proposed method is able to detect genomic regions which expected biases in gene expression, in this case on the basis of a cytogenetically invisible chromosomal aberration. (2) As in Figure 6, reducing *λ*_*N *_allows the algorithm to emphasize on larger regions, while suppressing smaller regions.

As illustrated in Figure 5 and Additional file 3, the TV method was able to identify numerous regions with biased expression in the specific leukemic subtypes. In broad outline, the key observations were as follows. In hyperdiploid ALL, each case exhibited elevated gene expression across one or more of the chromosomes 4, 6, 10, 14, 17, 18, 21 and X. This observation is consistent with the well-known fact that hyperdiploid ALL is characterized by extra copies of these chromosomes, and generally exhibits a total of more than 50 chromosomes (median 55). The finding is also consistent with previous studies indicating that a substantial proportion of the genes located on the gained chromosomes exhibit higher-than-expected expression levels on average [2,26]. In *TCF3/PBX1*-positive ALL, the most striking finding was that, in the majority of cases, a large region on 1q distal to the *PBX1 *locus was over-expressed whereas a small region (~1.6 Mb) on 19p distal to the *TCF3 *locus was under-expressed (Figure 6). These observations are in accordance with the fact that the *TCF3/PBX1 *fusion oncogene is the result a reciprocal translocation between chromosomes 1 and 19, where the translocated chromosome 19 is retained whereas the rearranged chromosome 1 is lost, followed by a reduplication of the normal chromosome 1 homologue [30]. In other words, the leukemic cells will exhibit a gain of 1q material and a loss of 19p material, where the latter aberration is usually cytogenetically invisible. In *ETV6/RUNX1*-positive ALL, recurrent changes in expression were observed in 6p22, 18q12, 21q22 and Xq25-28. Out of these, the over-expression over Xq25-28 was found to be particularly striking (Figure 7). Interestingly, this region was not known to be recurrently gained in *ETV6/RUNX1*-positive ALL until recently when, following more detailed aCGH-based investigations by us, the region was shown to be frequently duplicated [20]. In *MLL*-rearranged and *BCR/ABL1*-positive ALL, no convincing recurrent changes were found. Finally, in T-ALL, we observed numerous differentially expressed regions. The degree of differential expression in these regions was generally very high, suggesting that the underlying mechanism is regulatory rather than a gene-dose effect on the basis of underlying DNA copy number aberrations. Taken together, these results support that the described method is capable of identifying genomic regions with expectedly increased/decreased average gene expression, in the cases shown on the basis of imbalanced chromosomal aberrations (including examples of cytogenetically invisible changes).

For completeness, we note that detected segments corresponding to duplications and deletions display step heights around 0.5 to 1.0. Given that the variance of the gene scores is approximately one, this indicates that the SNRs used in the simulations are adequate (Materials and methods). We also note that the widths and heights of the smaller segments detected were in line with the resolutions predicted by Equation 7, supporting that this way of calculating the regularization parameters is reasonable. Finally, we remark that segmentation without prior normalization (except log-scale conversion) yielded poor results, verifying the necessity of using appropriately normalized gene scores (Materials and methods).

## Discussion

Genomic regions with altered gene expression arise in cancer cells because of acquired gains or losses of chromosomal material or epigenetic changes. The detection and delineation of such regions in gene expression maps relies on the availability of specialized segmentation techniques.

We have described a novel segmentation method based on TV minimization. The value of this method lies in that it combines significantly improved detection performance with an enhanced ability to delineate relevant regions. The explanation for these improvements is two-fold. First, adopting the TV norm as a regularity measure makes the segmentation procedure more robust under low SNRs. Previously, the TV norm has been successfully applied to numerous restoration problems in signal and image processing, including problems in bioinformatics [31]. Second, to extend further the performance of TV minimization, we have introduced a novel strategy for using multiple regularization parameters simultaneously. This feature allows for improved detection of regions with widely varying characteristics, while still allowing large regions to be detected without excessive over-segmentation.

Previously, other segmentation methods have been proposed. In contrast to our method, these are primarily tuned for aCGH or SNP array data, and perform less well under expression data-like conditions [24,25]. Similar to our method, a common theme is to fit piece-wise constant solutions to the data by dynamic programming under various goodness criteria, including penalized likelihood [22], penalized least squares [32], Bayesian posterior probability [33], edit distances [34] or hidden Markov models [35-37]. However, previous methods regularize the solution using a constant step penalty, impeding their performance on expression data. Other methods that are not based on dynamic programming but with similar behavior have been proposed [23,38-40], as have various smoothing methods [13,41-48]. The latter do not produce a segmentation, but, in some cases, tend to blur the edges between regions.

Using childhood ALL as an example, we have verified that our method is capable of identifying regions with increased/decreased expression on the basis of known chromosomal imbalances (including gross abnormalities as well as cytogenetically invisible aberrations). Previously, Callegaro et al [41] analyzed the Ross et al data set using an adaptive filtering approach. These authors found a differentially expressed region around the *PBX1 *locus on chromosome 1 in *TCF3/PBX1*-positive ALL, but did not report the footprints in expression of chromosomal imbalances revealed here. The Ross et al data were also studied by Hertzberg et al [2] who demonstrated the predictability of whole-chromosome gains in hyperdiploid ALL, but did not analyze the data at the sub-chromosomal level.

Technically, our scheme differs from the original TV scheme [21] in that we require the solution to be piece-wise constant instead of piece-wise continuous. The motivation for this restriction is four-fold. First, the piece-wise continuous model is less well suited for noisy conditions, partly because of its higher flexibility [49]. Second, a piece-wise constant signal model is natural in our application. Third, we achieve simultaneous de-noising and segmentation. Fourth, the globally optimal solution to the piece-wise constant TV minimization problem can be rapidly computed by dynamic programming.

The behavior of our method is controlled by the set of *λ *and the relevance threshold. Of note, we provide theory to calculate suitable *λ*, which hence can be regarded as more or less 'fixed'. Thus, the only parameter the user has to select is the relevance threshold. This parameter is easy to interpret.

Regarding possible improvements, we note that estimating *μ*_*i *_as the average of *f *over *I*_*i *_is reasonable when *π*_*i *_is near zero, but does not compensate for the fact that 'non-influenced genes' pull the estimate towards zero when *π*_*i *_is large. In principle, this artifact could be avoided by estimating *μ*_*i *_and *π*_*i *_using more advanced techniques, such as mixture-fitting. We have refrained from such extensions because of the anticipated computational overhead, and leave improvements in this direction as an open problem.

## Conclusion

In conclusion, we have described an enhanced methodology for identifying genomic regions with altered gene expression in cancer. Hence, this work, along with other efforts, should facilitate the search for genetic and epigenetic changes involved in cancer development.

## Materials and methods

### Problem definition

Let *f *(*x*) : *I *→ **R **be the gene expression score at chromosomal position *x *in some interval *I *(one such score is discussed below). This expression map can be regarded as a mixture of two signal components: a high-frequency component *v*(*x*) that corresponds to noise plus intrinsic variability in gene expression, and a low-frequency component *u*(*x*) that represents a more slowly varying gene expression bias profile. The segmentation problem can be formulated as the reconstruction of *u*(*x*) from *f *(*x*) subject to the constraint that *u*(*x*) is piece-wise constant, that is *u*(*x*) = *μ*_*i*_, *x *∈ *I*_*i *_for some plateau levels *μ*_*i *_∈ B and some set of ordered intervals *I*_1_, *I*_2_, ..., *I*_*M *_representing a disjoint partitioning covering *I *with a varying number of segments *M *(true number unknown *a priori*).

### Segmentation by piece-wise constant TV minimization

We propose to reconstruct *u *from *f *by solving the variational problem

(1) $$u^{*}=\min\int_{I}\left|u^{\prime }\right|+\lambda {\left(u-f\right)}^{2}dx,$$

where *u *is piece-wise constant. In the integrand, the first term is the *L*^1 ^norm of *u'*, also known as the TV norm of *u*. In this application, we interpret the TV norm literally, meaning that it is equal to the sum of the magnitudes of the steps in *u *(because *u' *is Dirac at the change points). Thus, it is clear that the role of the first term is to regularize the solution and prevent over-segmentation. By contrast, the second term is an *L*^2 ^fidelity term, or fitting term, serving to impose consistency with the original data and to counteract under-segmentation. The relative impact of the two competing terms is determined by the regularization parameter *λ *> 0. Trivially, *λ *= 0 produces the constant solution *u *= Average {*f*_*j*_}_*i *∈ *I *_(the most rigid model), whereas *λ *→ ∞ implies u* → f (the most flexible model). The choice of regularization parameter is discussed in detail in the following.

For computational tractability, we restrict the set of feasible solutions to the set of *u *such that ${\left\{\mu_{i}\right\}}_{1}^{M}$ is equal to the means of *f *over ${\left\{I_{i}\right\}}_{1}^{M}$. The benefit of this maneuver is that *u *will be uniquely defined for any partitioning, meaning that finding u* is reduced to finding an optimal partitioning of *I *which can be performed efficiently. The limitation is that we no longer account for the fact that the optimal *μ*_*i *_can be different from the mean over *I*_*i *_and there is strain on the solution.

To find the optimal solution to the restricted version of the problem, we convert from continuous to discrete form, adopting the notation *f*_*j *_= *f *(*x*_*j*_) where *x*_*j *_are the positions of probe *j *= 1, ..., *N *in ascending order along the chromosome. We obtain the discrete-form objective

(2) $$J(u)=\sum_{i=2}^{M}\left|\mu_{i-1}-\mu_{i}\right|+\lambda \sum_{i=1}^{M}\sum_{j\in I_{i}}{\left(\mu_{i}-f_{j}\right)}^{2},$$

where equal probe spacing has been assumed when discretizing the integral. The calculations can be modified to accommodate for unequally spaced probes if needed (not shown).

Next, we let *n *be an integer such that 1 ≤ *n *= *N *and $J_{n}^{*}$ the value of the objective function for the optimal segmentation of the (closed) integer interval [1, *n*]. Further, we let *n'*, 1 ≤ *n' *≤ *n *be the starting point for the last segment in that segmentation, and *n" *be the starting point of the last interval of the optimal segmentation of [1, *n'*]. Finally, we let the functions *μ*(*a*, *b*) and *ν *(*a*, *b*) denote the average and sum-of-squares about the average, respectively, of *f *over [*a*, *b*]. In this notation, Equation 2 reads

(3) $$J_{n}^{*}=\underset{n^{\prime }}{\min}\{J_{n^{\prime }-1}^{*}+\left|\mu (n^{\prime \prime },n^{\prime }-1)-\mu (n^{\prime },n)\right|+\lambda \nu (n^{\prime },n)\}.$$

Thus, given the optimal values of the objective function for the intervals [1, 1], [1, 2], ..., [1, *n *- 1], the optimal value of the objective for the next interval [1, *n*] can be computed explicitly. In other words, the problem satisfies the Bellman condition of optimality [50], and the inductive solution given by Equation 3 represents the forward pass of a dynamic programming scheme. For clarity, we give the details of the algorithm in pseudocode (Additional file 4). In its basic form, the algorithm is *O*(*N*^3^) in time and *O*(*N*) in memory. However, the time complexity can be reduced to *O*(*N*^2^) by loop unfolding (Additional file 4).

### Selection of regularization parameter

The traditional way to select *λ *in TV minimization is as follows. Let $\hat{u}$(*x*, *λ*) denote the *u*(*x*) estimate for a specific value of *λ*. If we assume for a while that the signal is purely additive (that is, *f *(*x*) = *u*(*x*) + *v*(*x*)), then the difference *f *(*x*) - $\hat{u}$(*x*, *λ*) will represent an estimate of the high-frequency component *v*(*x*) if *λ *has been chosen correctly. If, in addition, *v*(*x*) is independently and identically distributed with variance *σ*^2^, a natural constraint on *λ *is

(4) $$\text{Var}\{\hat{v}(x,\lambda )\}=\text{Var}\{f(x)-\hat{u}(x,\lambda )\}\approx \sigma^{2}.$$

This constraint is often used as a 'rule-of-thumb' stating that *λ *should be selected such that the variance of the residual $\hat{v}$(*x*, *λ*) is on par with *σ*^2^, which, in turn, can be estimated from the data or is known *a priori*.

While straightforward, the traditional way of selecting *λ *is inappropriate in our application. First, a purely additive signal model is too simplistic as some genes may not be influenced by the underlying genomic change. This undermines the validity of Equation 4. Second, it is diffcult to find a single *λ *that suits all region sizes simultaneously. Small *λ *allow the baseline to 'break up', and hence allow small regions to be detected. At the same time, however, relevant regions will become over-segmented (fragmented), which is undesirable. Conversely, large *λ *values will keep large regions intact, whereas small regions cannot be identified.

To alleviate this issue, we developed an extended TV minimization-based scheme that allows a range of *λ *values to be used simultaneously, as opposed to relying on a single value. We proceeded as follows. First, let *λ*_1 _<*λ*_2 _< ⋯ <*λ*_*N *_be an increasing sequence of *λ *values, and *T*_*μ *_a threshold that specifies the smallest plateau level |*μ*_*i*_| that is required for a segment to be called 'relevant'. Second, segment *I *using *λ*_1 _as regularity parameter (most rigid model). Third, mark all segments that satisfy *μ*_*i *_> *T*_*μ *_as finished and exclude them from further processing. Fourth, the subintervals that are 'non-relevant', that is whose |*μ*_*i*_| do not exceed *T*_*μ *_are re-segmented using *λ*_2 _(slightly less rigid model). The process is repeated with *λ*_3_, *λ*_4_, and so on until *λ*_*N *_is reached, or no more 'non-relevant' regions remain. In other words, the segmentation scheme first searches for large, relevant regions and then proceeds to search for successively smaller regions in a recursive manner. Thus, the algorithm allows large relevant regions to be detected without excessive over-segmentation, while still allowing smaller regions to be detected.

To obtain guidance for the selection of the *λ *series, we considered the following hypothetical case. Assume that the gene expression map of *I *is non-biased everywhere, except in a contiguous region *I' ⊂ I *where gene expression is biased (that is, the mean gene score is non-zero). Let $J^{0}$ denote the value of the total variation functional (Equation 1) for the one-segment solution *u*(*x*) = 0 for all *x *∈ *I*, including *I'*.

Similarly, let $J^{1}$ denote the value of the functional for a three-segment solution where *u*(*x*) is zero when *x *∈ *I\I' *but the average of *f*_*j *_over *I *when *x *∈ *I '*. By Equation 1, we have

(5) $$J^{0}-J^{1}=\lambda \sum_{i\in I^{\prime }}f_{j}^{2}-\left(\frac{2}{N^{\prime }}\left|\sum_{i\in I^{\prime }}f_{j}\right|+\lambda \sum_{i\in I^{\prime }}f_{j}^{2}-\lambda \frac{1}{N^{\prime }}{\left(\sum_{i\in I^{\prime }}f_{j}\right)}^{2}\right),$$

where *N' *is the number of genes in *I'*. The right-hand side simplifies to

(6) $$-\frac{2}{N^{\prime }}\left|\sum_{i\in I^{\prime }}f_{j}\right|+\frac{\lambda }{N^{\prime }}{\left(\sum_{i\in I^{\prime }}f_{j}\right)}^{2},$$

which is positive if and only if

(7) $$\lambda >2\cdot \frac{1}{\left|\sum_{i\in I^{\prime }}f_{j}\right|}.$$

This inequality provides a criterion for determining when the (more correct) three-segment solution will be preferred over the (too rigid) one-segment solution. The inequality states that, for the three-segment solution to be selectable, *λ *must exceed a bound that is inversely proportional to the sum of the *f*_*j *_in *I'*. The latter in turn is approximately proportional to the width of *I' *and the expectancy of *f*_*j *_(assuming that the *f*_*j *_are similarly distributed in *I'*) on average.

This criterion can be used to identify reasonable *λ *values. First, the simplest segmentation result is a one-segment solution that spans the entire chromosome. This solution will be guaranteed to be tested for if *λ*_1 _= 0. Second, *λ*_*N *_specifies the most flexible model used. For this parameter, we used the value 2/5. This choice allows for the resolution of segments with |∑*f*_*j*_| around 5, a level that corresponds to, for example, a 5-probe segment with plateau level around 1.0, or a 10-probe segments with a plateau level around 0.5. Given the characteristics of the data (noisy, low genomic resolution and the presence of 'non-influenced' genes), we cannot hope to detect segments with very few probes, making this choice of *λ*_*N *_reasonable. In this context, we remark that if the aim is to detect individual differentially expressed genes, several dedicated methods are available for this purpose (for example, *t *statistic-based approaches). Third, it remains to select *λ *values between *λ*_1 _and *λ*_*N*_. It seems natural to select *λ *that correspond to equally, and sufficiently densely, spaced ∑*f*_*j *_. Taken together, these considerations motivate the series *λ*_1 _= 0, *λ*_2 _= 2/100, *λ*_3 _= 2/99, ..., *λ*_*N *_= 2/5, which was used both in the simulations and on real data.

Apparently, this series can be altered by changing *λ*_*N *_and the density of in-between values. We illustrate the effect of changing *λ*_*N *_by using two truncated series (*λ*_*N *_= 2/30 and *λ*_*N *_= 2/15) in the Results section. We also repeated the experiments with twice as dense and twice as sparse *λ*, yielding results in broad agreement with those presented (data not shown). The choice *λ*_1 _= 0 is not subject to tweaking, as we will always be interested in knowing whether the one-segment (whole-chromosome) solution is relevant.

### Simulation model

In the simulation study, we generated artificial chromosomes by mixing known, piece-wise constant expression bias profiles with a randomly generated high-frequency signal component (corresponding to noise plus inherent variability in gene expression). Within the *i*th segment, we regard the *f*_*j *_as following the mixture distribution $f_{j}\sim \pi_{i}N(0,\sigma^{2})+(1-\pi_{i})N(\mu_{i},\sigma^{2})$, where *μ*_*i *_is the plateau level, *σ*^2 ^variance of the noise and variability in expression around the baseline and *π*_*i *_is the proportion of non-influenced genes, that is, the percentage of genes whose expression is not altered by the underlying genetic alteration. The reason for using a mixture distribution is to allow the model to accommodate for genes that are completely transcriptionally inactive or are targeted by 'bad' probes.

In the first two simulation experiments (ROC and breakpoint distributions), we used expression profiles with central square-wave step of varying width and height. In this case, the simulation model has four parameters: the chromosome length, the aberration width, the SNR and the proportion of non-influenced genes (*π*). For tractability, we assume the same proportion of non-influenced genes in all segments.

The parameter values were as follows. The chromosome length was fixed to 100. However, to verify robustness, the experiments were repeated with numerous other values, yielding results in agreement with those presented (data not shown). The aberration widths were 10, 20 or 40. The SNRs were 0.5, 1.0 and 2.0. Roughly, the first two values correspond to expression data-like conditions, whereas the last value corresponds to aCGH data-like conditions. The proportions of non-influenced genes were 0.0, 0.1, ..., 0.5, ranging from regions with few non-influenced genes to regions with large proportions of non-influenced genes. When *π *= 0.0, our simulation model is identical to that used in [24].

### Performance assessment measures

To assess the performance of the different algorithms, we calculated ROC curves and breakpoint maps for all combinations of aberration widths, SNRs, and proportions of non-influenced genes. To generate ROC curves, we calculated the TPRs and the FPRs across 200 simulated chromosomes as we varied the threshold for determining the relevance of an aberration. TPRs were calculated as the number of genes inside the central (biased) region whose segmented values are above the threshold level divided by the number of genes in the aberration. FPRs were defined as the number of genes outside the central region whose segmented values are above the threshold level divided by the total number of genes outside the aberration. To compute the ROC and false discovery rate curves, we varied the threshold for calling regions relevant from zero to the maximum gene score. Each threshold value yields a TPR and a FPR, represented by a point on the ROC curve. As noted in [24], TPR and FPR are informative in understanding how an algorithm performs in estimating the boundary of the altered region: when the algorithm over-estimates the boundary, FPR increases while TPR remains fixed; when it under-estimates the boundary, TPR decreases while FPR remains fixed.

To generate breakpoint maps, we simulated and segmented 10,000 chromosomes. We then counted the number of breakpoints identified at each chromosomal position and, finally, normalized the resulting histogram by dividing the value in each bin by the total number of breakpoints.

### Selection of parameter values in the control methods

When running the control methods DNAcopy and CGHseg, we used the default method parameters suggested in the original publications [22,23]. The motivation for this decision is three-fold. First, the use of default parameters is in agreement with previous comparative reviews, in which default parameters are used throughout [24]. Second, it can be argued that default parameters produce a realistic test scenario, as these are the parameters many users would use in practice [24]. Third, and finally, it appears reasonable to expect that default parameters have been made default because they produce good results in many cases. For completeness, however, we performed complementary experiments in which we used a broad range of other method parameter values, distinct from the default settings. These experiments support that the default parameters are in fact an appropriate choice (exemplified in Additional file 5).

### Microarray data and preprocessing

For testing, we used the data set by Ross et al [26] (obtained from [51]) containing expression profiles (Affymetrix U133A+B arrays) from patients with childhood ALL of different subtypes (Table 1). The expression values (log-scale) of each case were converted to *z*-scores with respect to all out-of-class cases, that is, for gene *j *in a given case, we let *f*_*j *_= (*y*_*j*_*- m*_*j*_)/*s*_*j*_, where *y*_*j *_is the expression value, *m*_*j *_the average expression value across cases whose classes differ from that of the case being analyzed and *s*_*j *_is the pooled within-class standard error given by Smyth's robust empirical Bayes estimator [52]. In principle, this type of data normalization is largely similar to the normalization performed when extracting lists of differentially expressed genes (by the use of *t*-statistics), although, in our case, we perform the normalization on a case-versus-group, rather than group-versus-group, basis.

The advantage of computing differential expression with respect to a pool of expression profiles from multiple tumor classes is that class-specific changes in expression will be diluted. In particular, we obtain a reference group that is approximately euploid on average, except in (hopefully uncommon) regions sharing copy number changes shared by all leukemic subtypes. We note that in cases when other tumor types are unavailable as controls, other relevant control samples can be used in their place. For instance, it may sometimes be natural to use expression profiles of normal tissue as reference profiles. We also note that *σ*^2 ^≈ 1 for all genes because of the definition of the *z*-score and the fact that most genes are non-differentially expressed between classes. This motivates the use of normal distributions with unit variance in the simulations.

In the experiments, we included the data set by Mullighan et al [29], consisting of DNA copy number profiles (Affymetrix 250 k SNP arrays) from the same types of tumors as those covered in the Ross et al data set. While the two data sets were produced by the same research group, the annotation provided does not allow copy number profiles to be paired with their corresponding expression profiles. Hence, the Mullighan et al and Ross et al data cannot be correlated case-by-case. However, by computing the average DNA copy number profile across each leukemic subtype, the Mullighan et al data could be used to obtain a map of common gains and losses of chromosomal material in specific ALL subtypes (see Results and Figure 5).

### Software availability

The described method has been implemented as a software package (Rendersome), which is freely available on request from the authors.

## List of abbreviations

aCGH, array comparative genome hybridization; ALL, acute lymphoblastic leukemia; SNP, single nucleotide polymorphism; SNR, signal-to-noise ratio; TV, total variance.

## Authors' contributions

BN developed the algorithm, designed and performed the experiments and wrote the manuscript. MJ participated in designing the algorithm and co-authored the manuscript. AH, SN and TF oversaw the experiments and co-authored the manuscript. All authors read and approved the final version of the manuscript.

## Additional data files

Additional data file 1 is a complete set of ROC curves from the simulation study.

Additional data file 2 is a complete set of breakpoint distribution plots from the simulation study.

Additional data file 3 is a high-resolution versions of the expression maps for each of the ALL cases in the Ross et al data set, as reconstructed by the proposed algorithm.

Additional data file 4 contains additional information about the proposed segmentation algorithms, including pseudocode. 

Additional data file 5 contains additional data illustrating that the use of the default method parameters for CGHseg is appropriate.

## Supplementary Material

Additional file 1 Complete set of ROC curves from the simulation study Click here for file

Additional file 2 Complete set of breakpoint distribution plots from the simulation study Click here for file

Additional file 3 High-resolution versions of the expression maps for each of the ALL cases in the Ross et al data set, as reconstructed by the proposed algorithm. Gray: Original gene scores from the Ross data set. Blue solid: Segmented gene scores. Each row represents one case, except for the bottom row which displays the average segmentation result and DNA copy number profiles (orange) across each leukemic subtype. Click here for file

Additional file 4 Additional information about the proposed segmentation algorithms, including pseudocode. Click here for file

Additional file 5 Additional data illustrating that the use of the default method parameters for CGHseg is appropriate. Click here for file
