# Supplementary material for: An improved method for detecting and delineating genomic regions with altered gene expression in cancer
Source: Genome Biol. 2008 Jan 21;9(1):R13. doi: 10.1186/gb-2008-9-1-r13 (PMC2395254; doi:10.1186/gb-2008-9-1-r13)
Supplement: Additional file 1 — Complete set of ROC curves from the simulation study [file gb-2008-9-1-r13-S1.pdf]

Additional file 1

This document contains the full set of receiver operating characteristics (ROC) curves from the simulation study. The generation and interpretation of these results is described in the main paper.

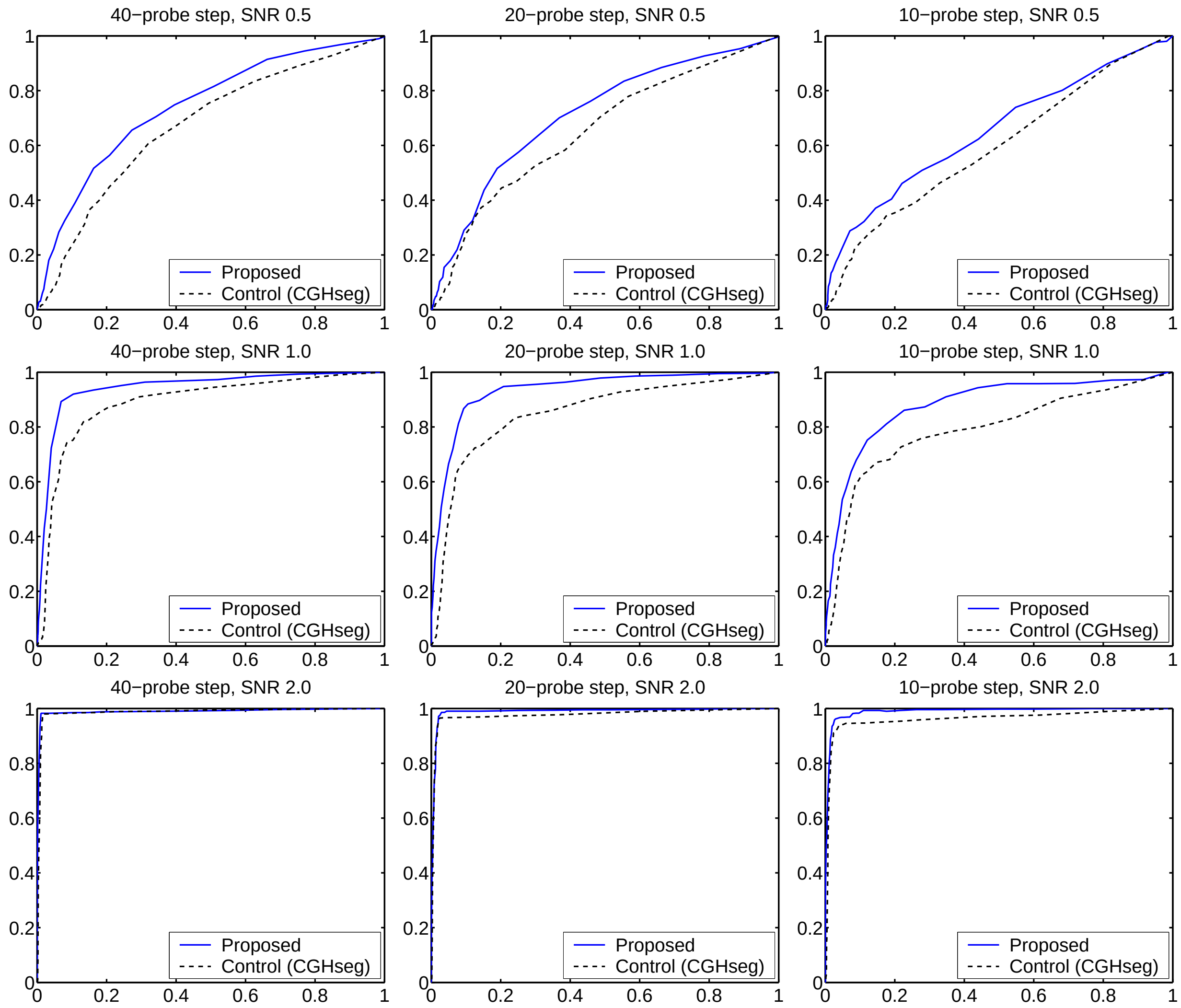

Receiver operating characteristics (ROC), $\pi = 0.0$

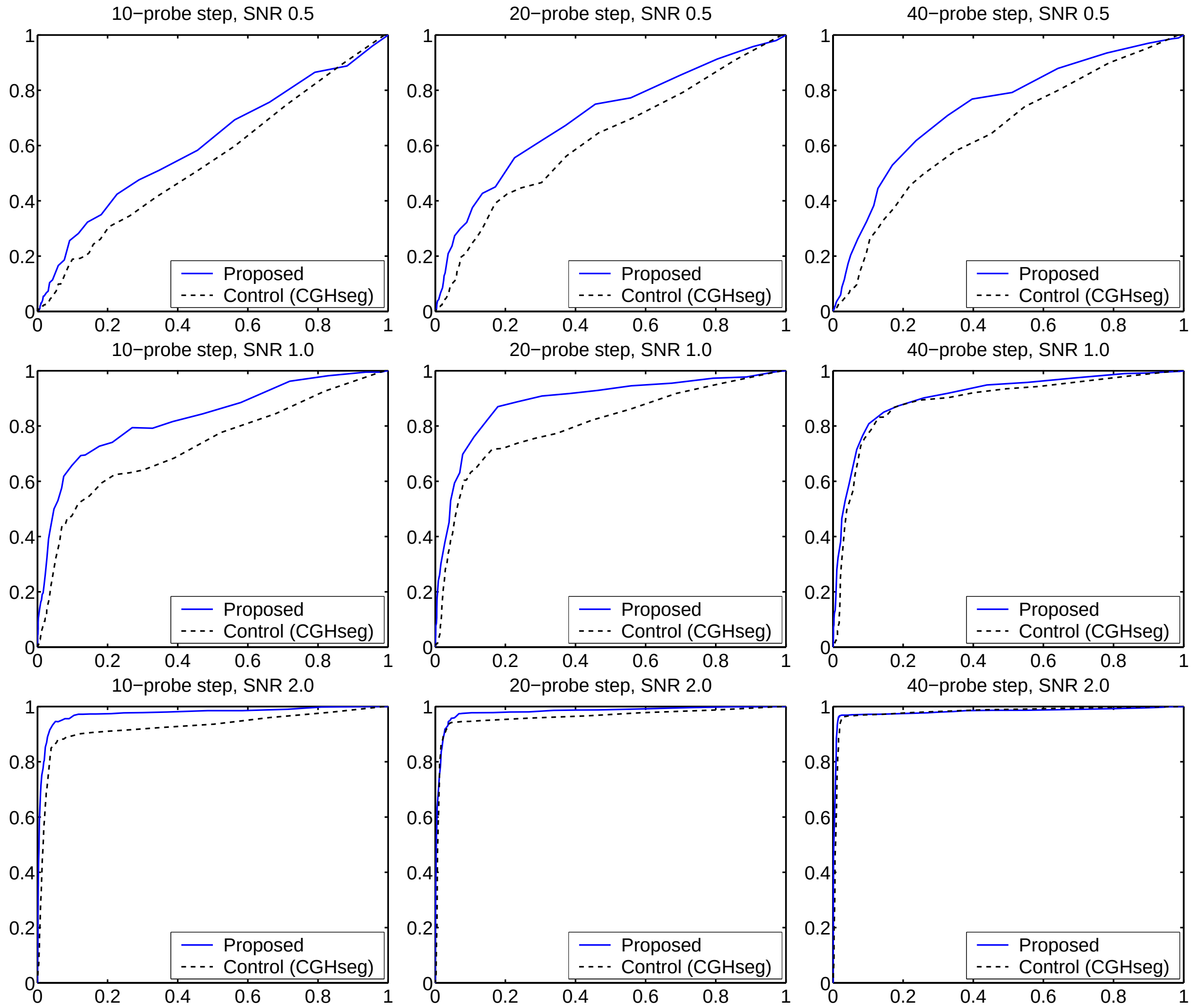

Receiver operating characteristics (ROC), $\pi = 0.1$

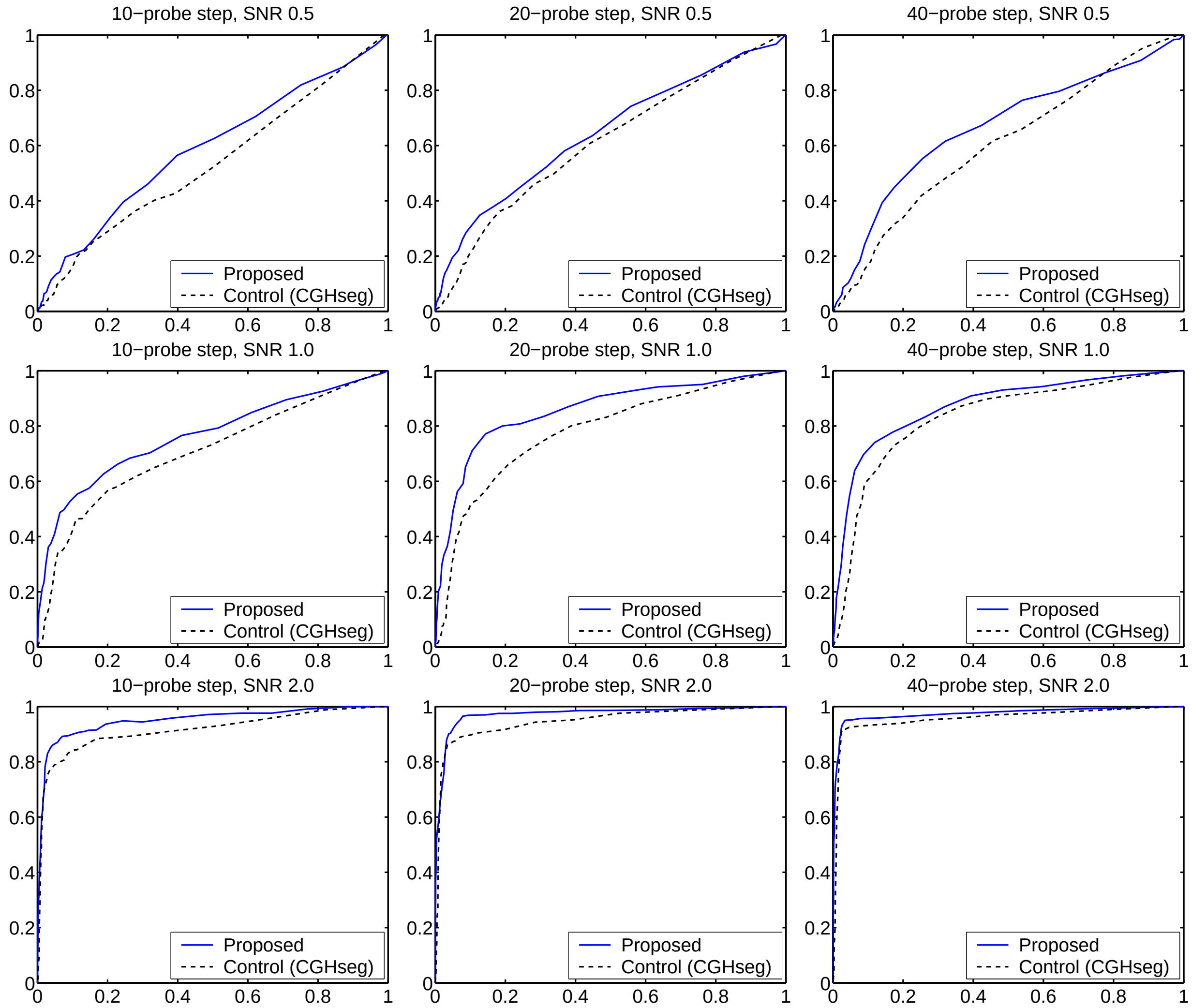

Receiver operating characteristics (ROC), $\pi = 0.2$

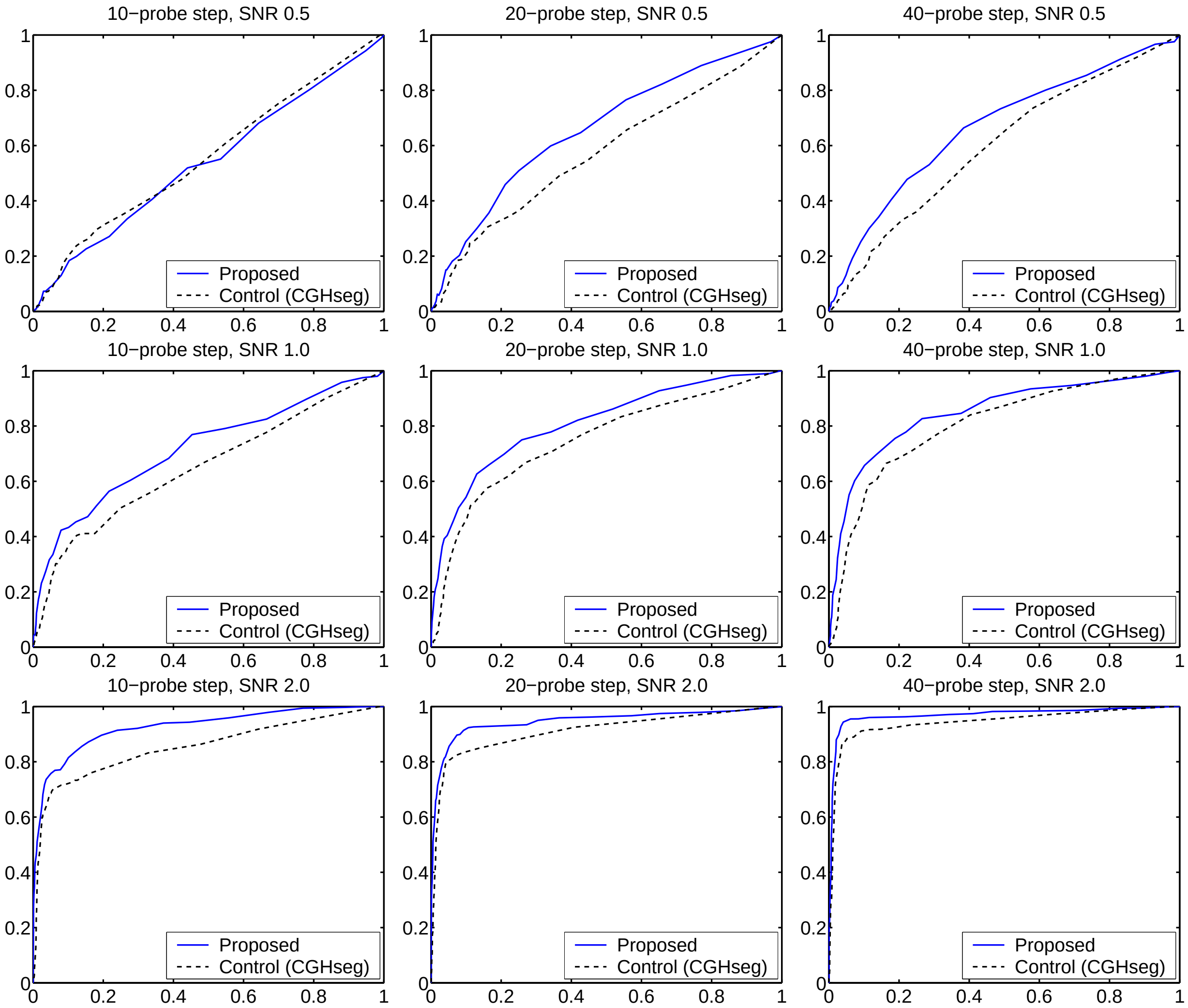

Receiver operating characteristics (ROC), $\pi = 0.3$

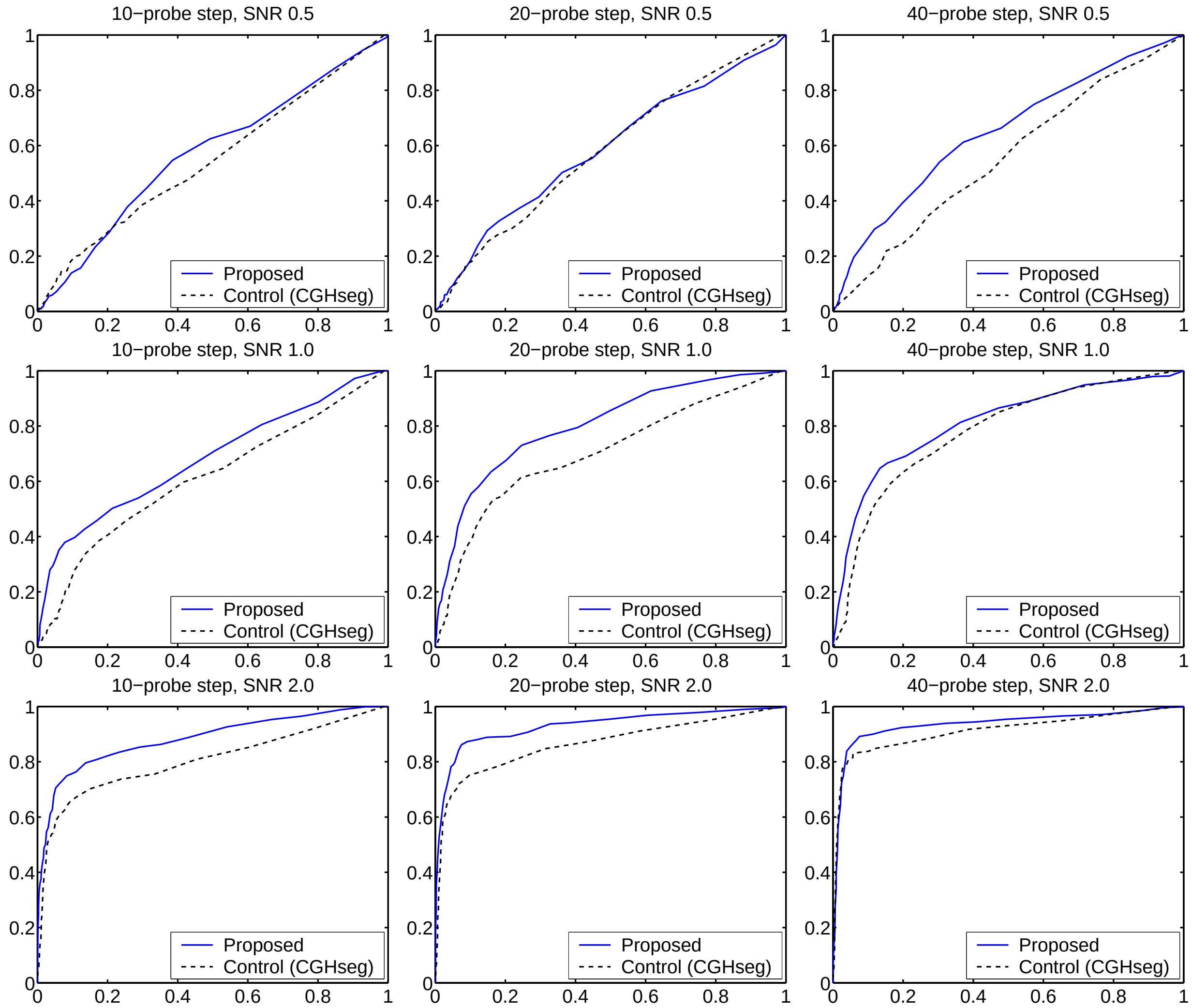

Receiver operating characteristics (ROC), $\pi = 0.4$

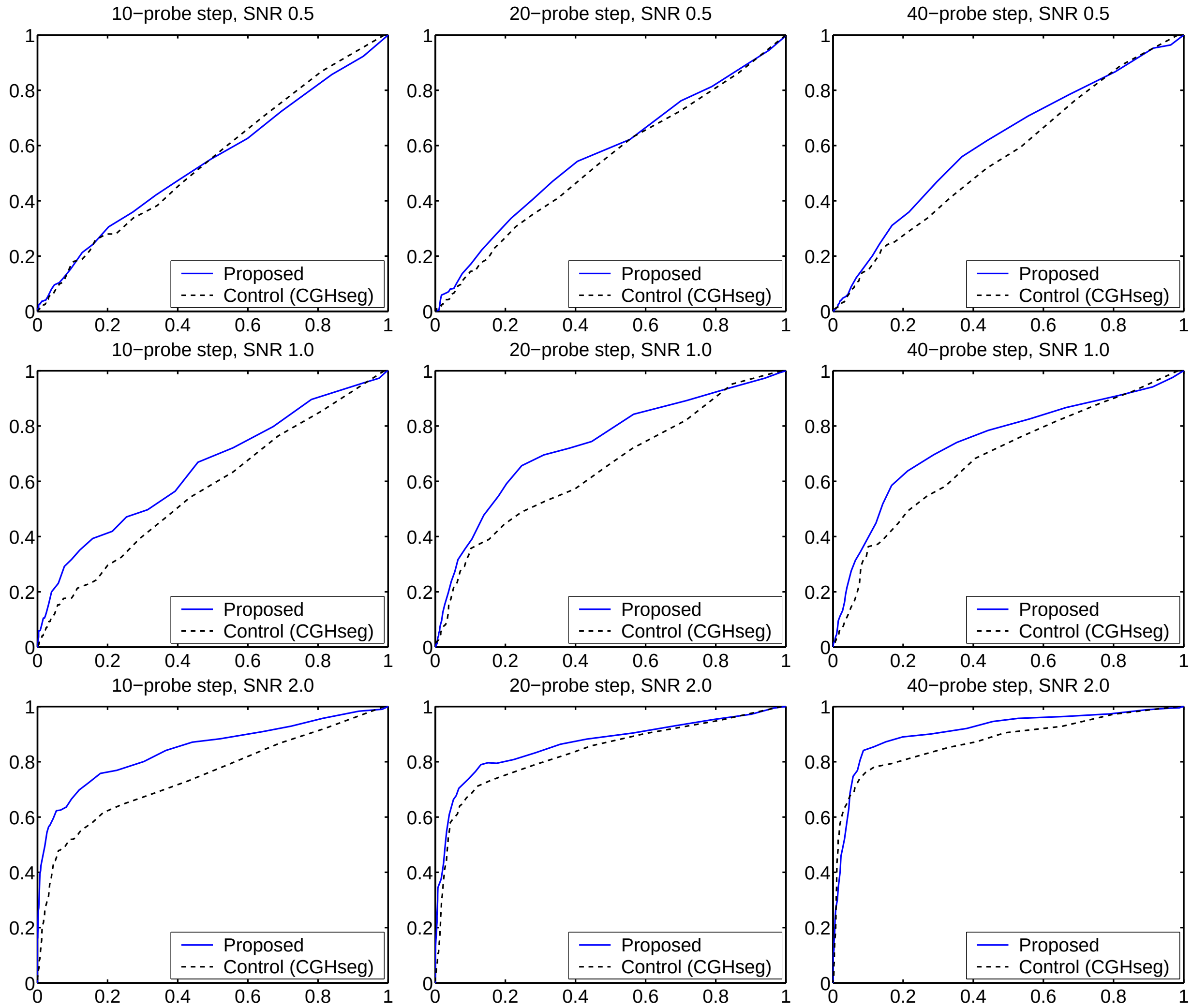

Receiver operating characteristics (ROC), $\pi = 0.5$
