# Supplementary material for: An improved method for detecting and delineating genomic regions with altered gene expression in cancer
Source: Genome Biol. 2008 Jan 21;9(1):R13. doi: 10.1186/gb-2008-9-1-r13 (PMC2395254; doi:10.1186/gb-2008-9-1-r13)
Supplement: Additional file 2 — Complete set of breakpoint distribution plots from the simulation study [file gb-2008-9-1-r13-S2.pdf]

Additional file 2

This document contains the full set of breakpoint distribution plots from the simulation study. The generation and interpretation of these results is described in the main paper.

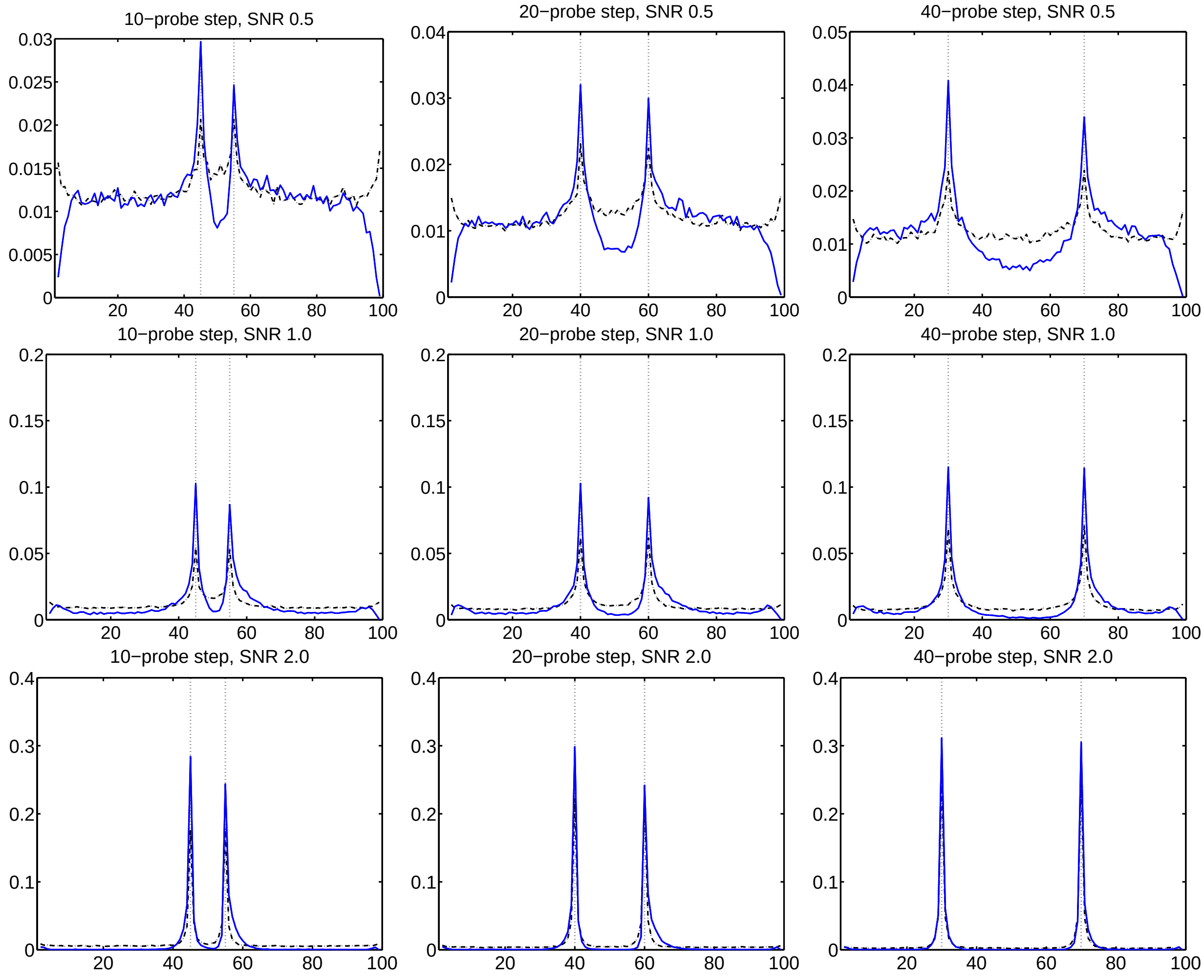

Breakpoint distribution, $\pi = 0.0$

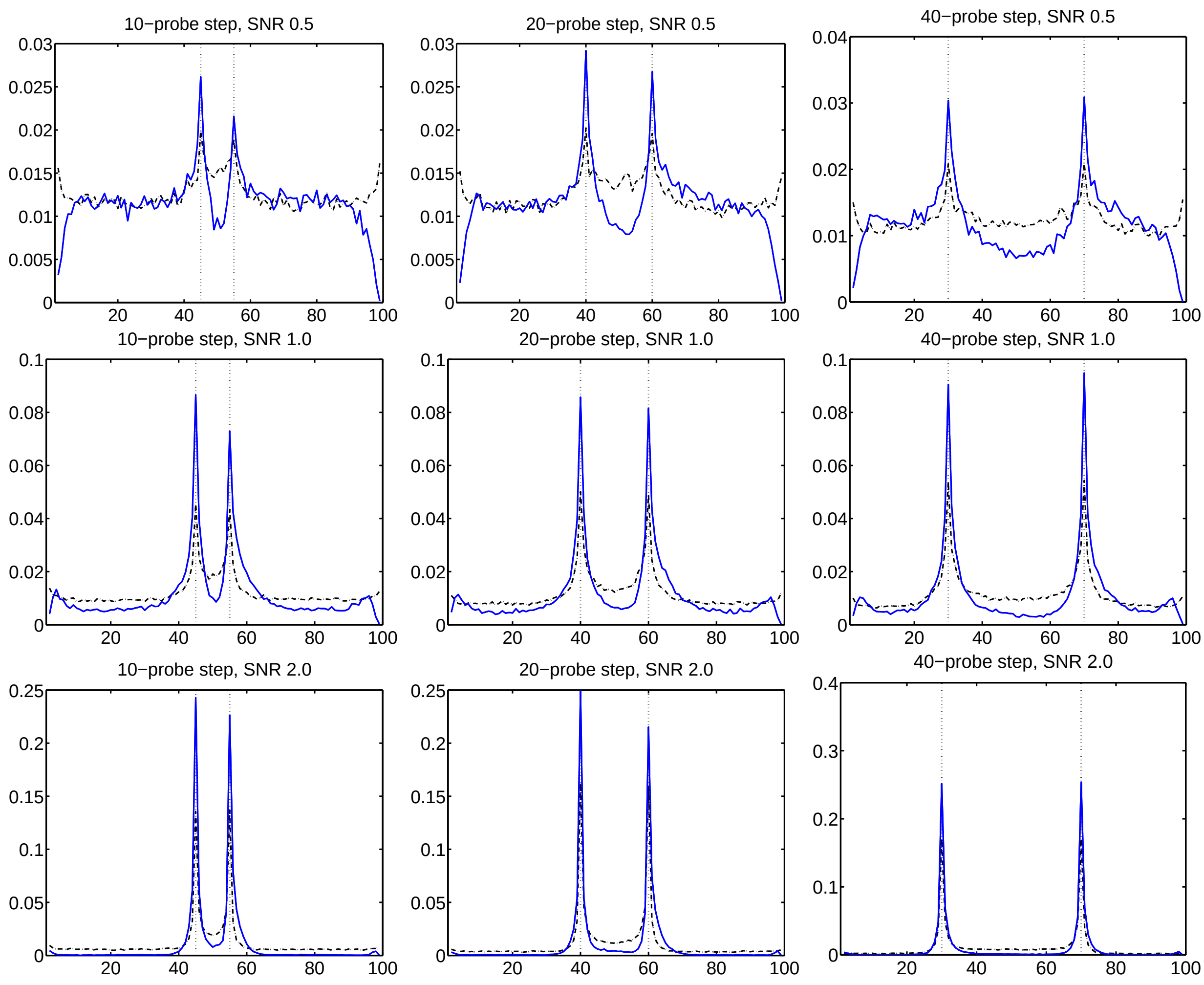

Breakpoint distribution, $\pi = 0.1$

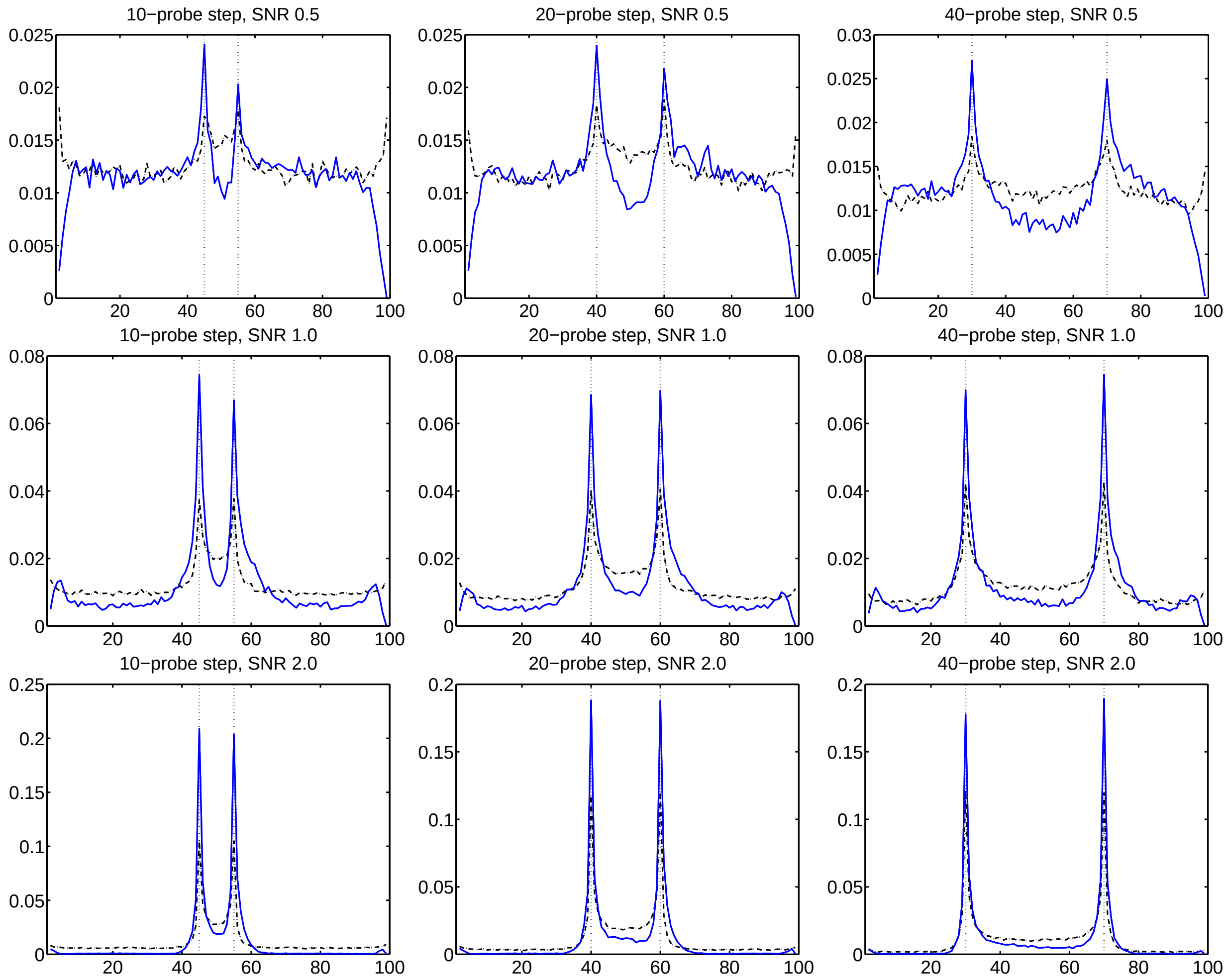

Breakpoint distribution, $\pi = 0.2$

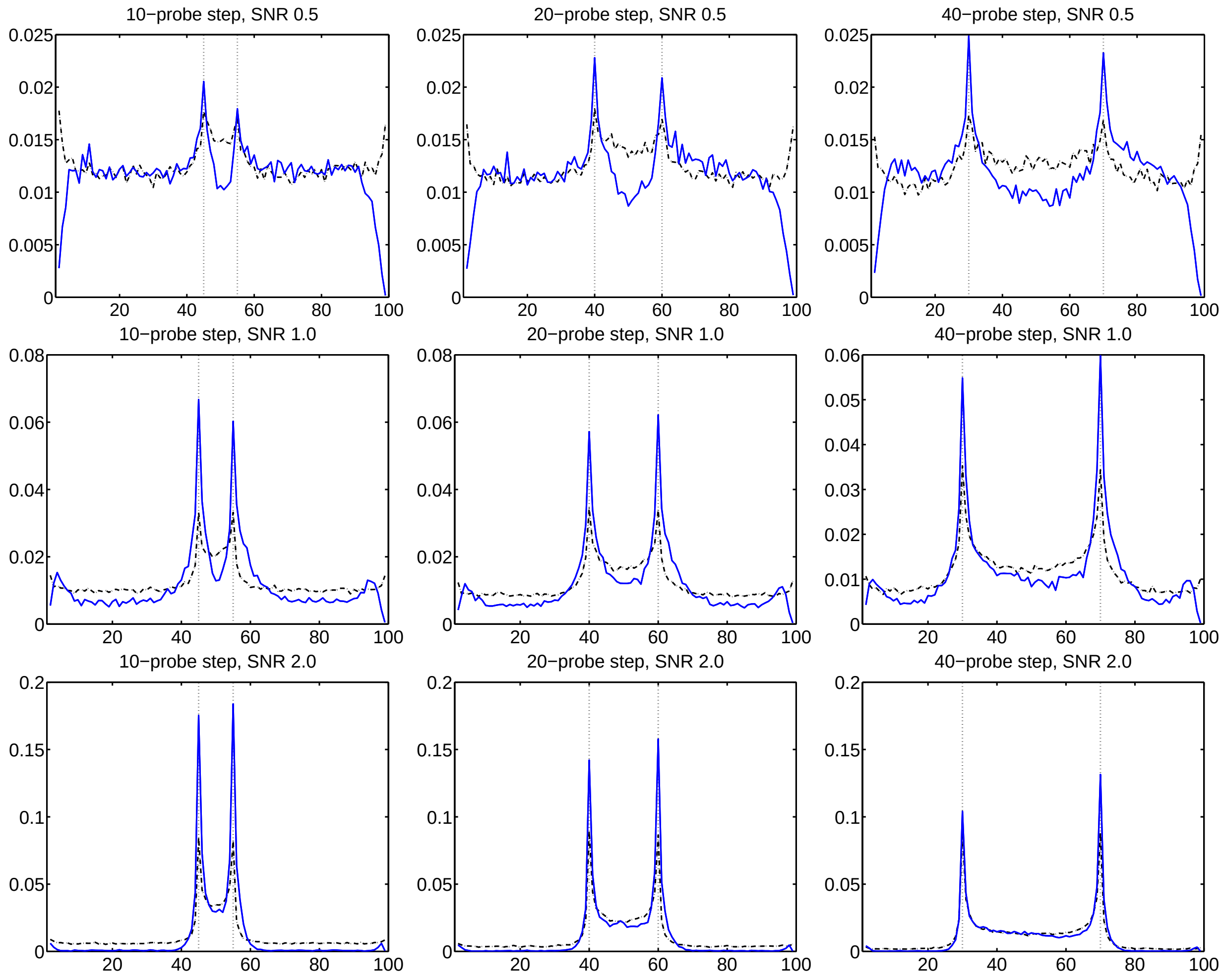

Breakpoint distribution, $\pi = 0.3$

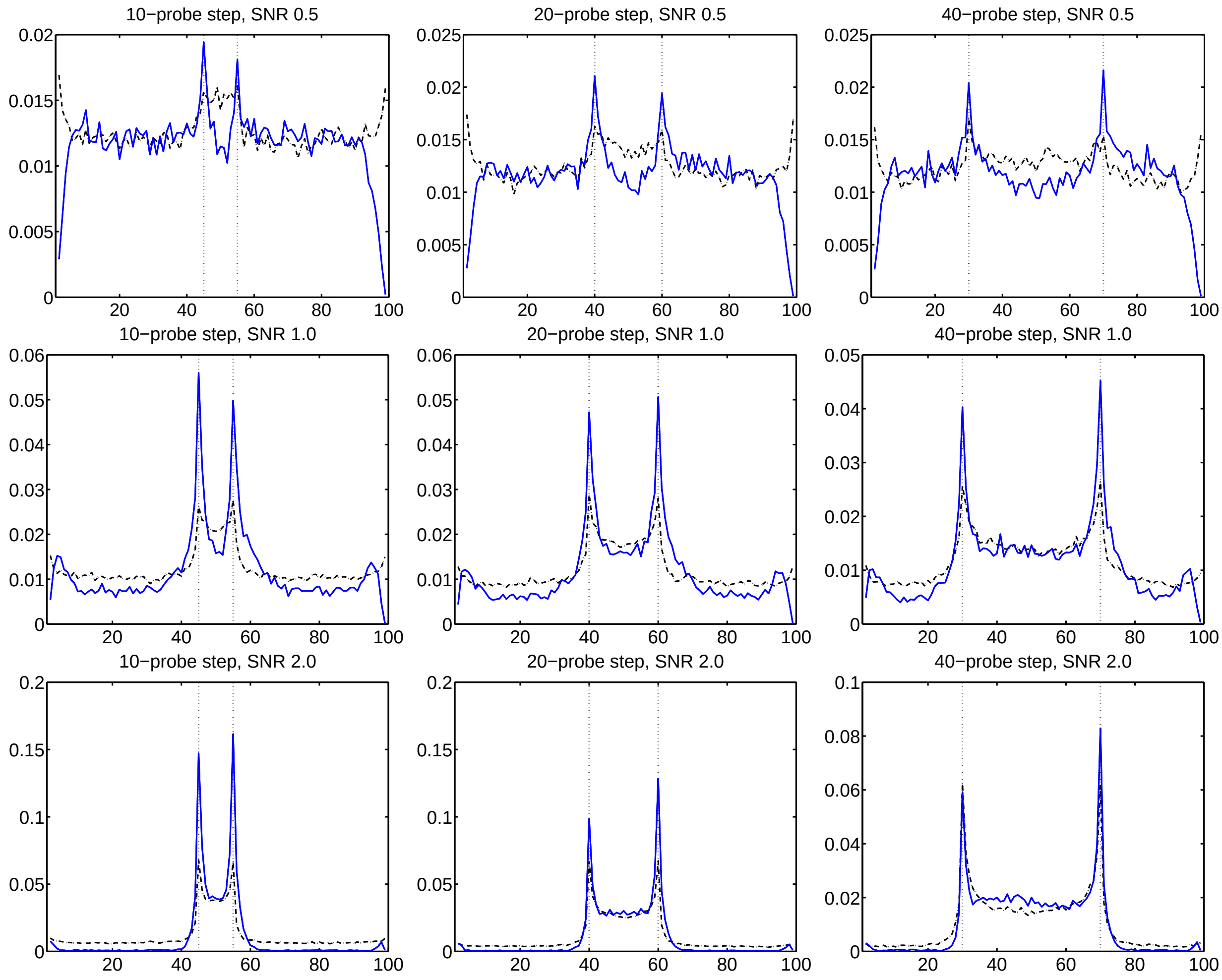

Breakpoint distribution, $\pi = 0.4$

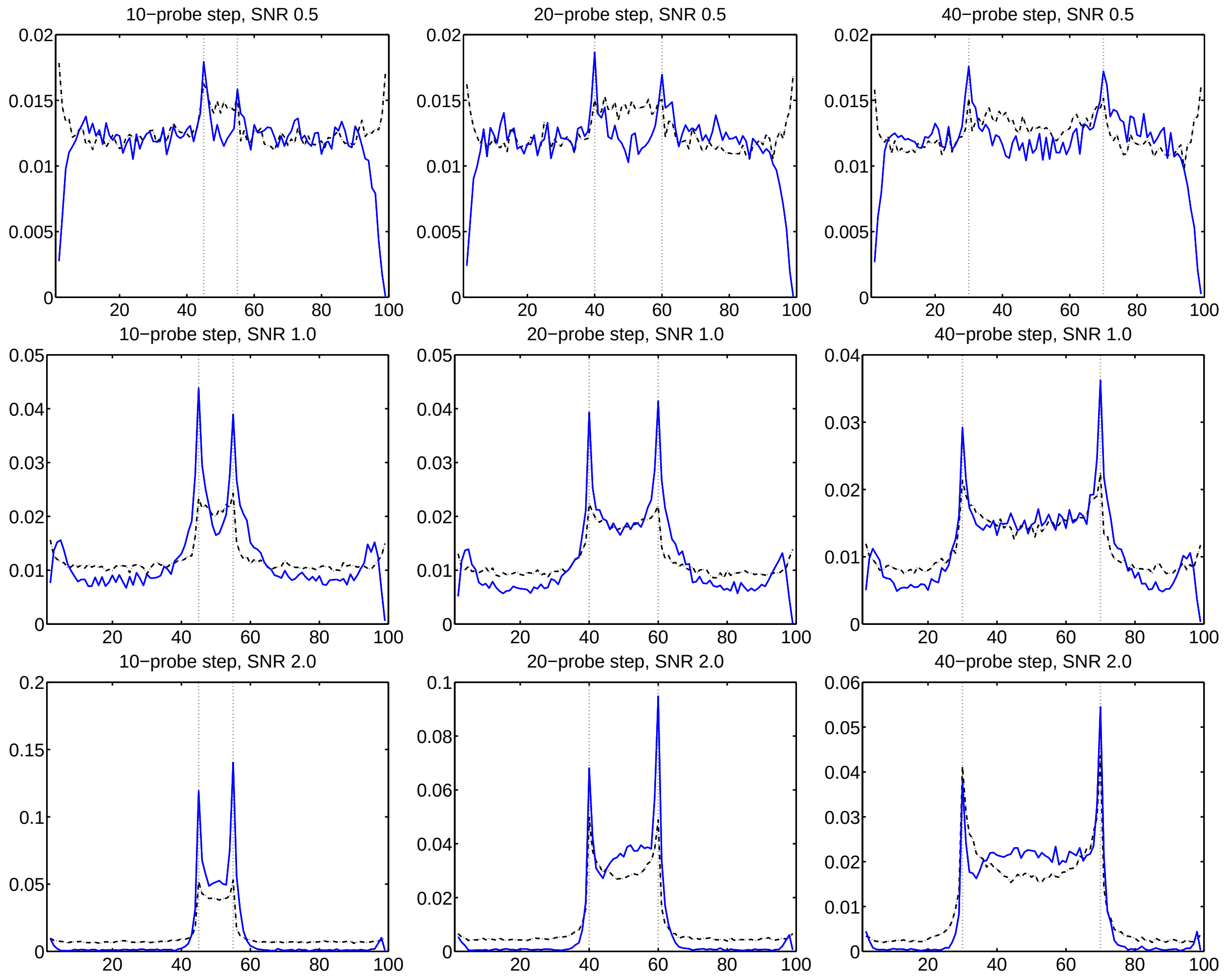

Breakpoint distribution, $\pi = 0.5$
