# Supplementary material for: An improved method for detecting and delineating genomic regions with altered gene expression in cancer
Source: Genome Biol. 2008 Jan 21;9(1):R13. doi: 10.1186/gb-2008-9-1-r13 (PMC2395254; doi:10.1186/gb-2008-9-1-r13)
Supplement: Additional file 4 — Additional information about the proposed segmentation algorithms, including pseudocode. [file gb-2008-9-1-r13-S4.pdf]

Basic algorithm in code

Let vF, vM, and vN be 1-based (*i.e.*, indexed by 1, 2, and so forth) vectors of size N where vF[n] stores the n:th gene score, vM[n] the average score across the last segment in the optimal segmentation of [1, n], and vN[n] the endpoint of the last-but-one segment of the optimal segmentation of [1, n]. Further, let vJ be a 0-based vector of size $N+1$ containing the optimal values of the objective function. This vector is initialized to "infinity" at all elements, except the first which is initially zero. In pseudocode, the forward dynamic programming pass is

```
// Forward pass. Basic version.
for (int n=1;n<=N;n++) {
    for (int n'=1;n'<=n;n'++)
    {
        s= 0;
        ss= 0;
        for (int j=n';j<=n;j++)
        {
            s  += vF[j];
            ss += vF[j]*vF[j];
        }
        mu= s/(n-n'+1);
        nu= (ss-s*mu);

        if (n'>1)
            // Modify mu_diff expression to adapt to L^0 or L^p norms
            mu_diff= abs(vM[n'-1] - mu);
        else
            mu_diff= 0;

        J= vJ[n'-1] + mu_diff + lambda*nu;
        if (J < vJ[n])
        {
            vJ[n]= J;
            vM[n]= mu;
            vN[n]= n'-1;
        }
    }
}
```

After the forward pass, the segmental breakpoints are recovered by backtracking through vN. The technical details follow the standard dynamic programming backtracking procedure and are therefore left to the reader.

Reducing the computational complexity

The forward dynamic programming pass considers $O(N^2)$ intervals, and spends $O(N)$ additions and multiplications on computing sums and sums-of-squares over each one. Thus, the overall time complexity is $O(N^3)$. However, by noting that the innermost loop can be unfolded by computing sums and sums-of-squares cumulatively, we arrive at the following considerably faster algorithm which is $O(N^2)$ in time:

```
// Forward pass. Fast version.
s_outer = 0;
ss_outer= 0;
for (int n=1;n<=N;n++)
{
    s_outer  += vF[n];
    ss_outer += vF[n]*vF[n];
    s_inner = s_outer;
    ss_inner= ss_outer;

    for (int n'=1;n'<=n;n'++)
    {
        mu= s_inner/(n-n'+1);
        nu= (ss_inner-s_inner*mu);
        if (n'>1)
            // Modify mu_diff expression to adapt to L^0 or L^p norms
            mu_diff= abs(vM[n'-1] - mu);
        else
            mu_diff= 0;

        J= vJ[n'-1] + mu_diff + lambda*nu;
        if (J < vJ[n])
        {
            vJ[n]= J;
            vM[n]= mu;
            vN[n]= n'-1;
        }

        s_inner  -= vF[j];
        ss_inner -= vF[j]*vF[j];
    }
}
```
