# Supplementary material for: An improved method for detecting and delineating genomic regions with altered gene expression in cancer
Source: Genome Biol. 2008 Jan 21;9(1):R13. doi: 10.1186/gb-2008-9-1-r13 (PMC2395254; doi:10.1186/gb-2008-9-1-r13)
Supplement: Additional file 5 — Additional data illustrating that the use of the default method parameters for CGHseg is appropriate. [file gb-2008-9-1-r13-S5.pdf]

Additional File 5

As discussed in Materials and Methods, we used default method parameter values for generating the CGHseg results shown in the simulation study. In this supplement, we provide complementary data supporting that the default values (suggested in Picard et al, 2005) are an appropriate choice.

In short, CGHseg performs segmentation by fitting piece-wise constant solutions with different numbers of segments to the data by minimizing an $\mathcal{L}^2$ fidelity term by dynamic programming. The algorithm first computes a range of solutions with different numbers of segments, and then employs a likelihood-based criterion to select adaptively the final number of segments. The main parameters are the maximum number of segments ($K_{\max}$) and a parameter called s that is used when selecting the number of segments. The method also offers a choice between a homoscedastic fitting model (equal variance for the high-frequency component in all segments) and a heteroscedastic fitting model (different variances in different segments). The default values suggested in the original work are $K_{\max} = 20$, $s = -0.5$, homoscedastic model (Picard et al, 2005).

To verify that the default parameters are in fact an appropriate choice, and hence to ascertain the validity and robustness of the conclusions of the simulation study, we run CGHseg with a broad range of method parameter values (distinct from the default values). We thus repeated the simulation experiments with numerous combinations of $K_{\max}$ and s, and with both the heteroscedastic and homoscedastic models. As exemplified in Figure 1 and Figure 2 below, many parameter choices (typically values near the default values) produced results on par with – but not substantially better than – those obtained with the default parameters. For other parameter choices (typically extreme or degenerate values), CGHseg performed less well than with the default parameters. Taken together, these data support that the CGHseg results presented in the main article can be regarded as reflecting the method performing at (or at least near) its best, making the comparison with the proposed (TV-based) method valid and sound.

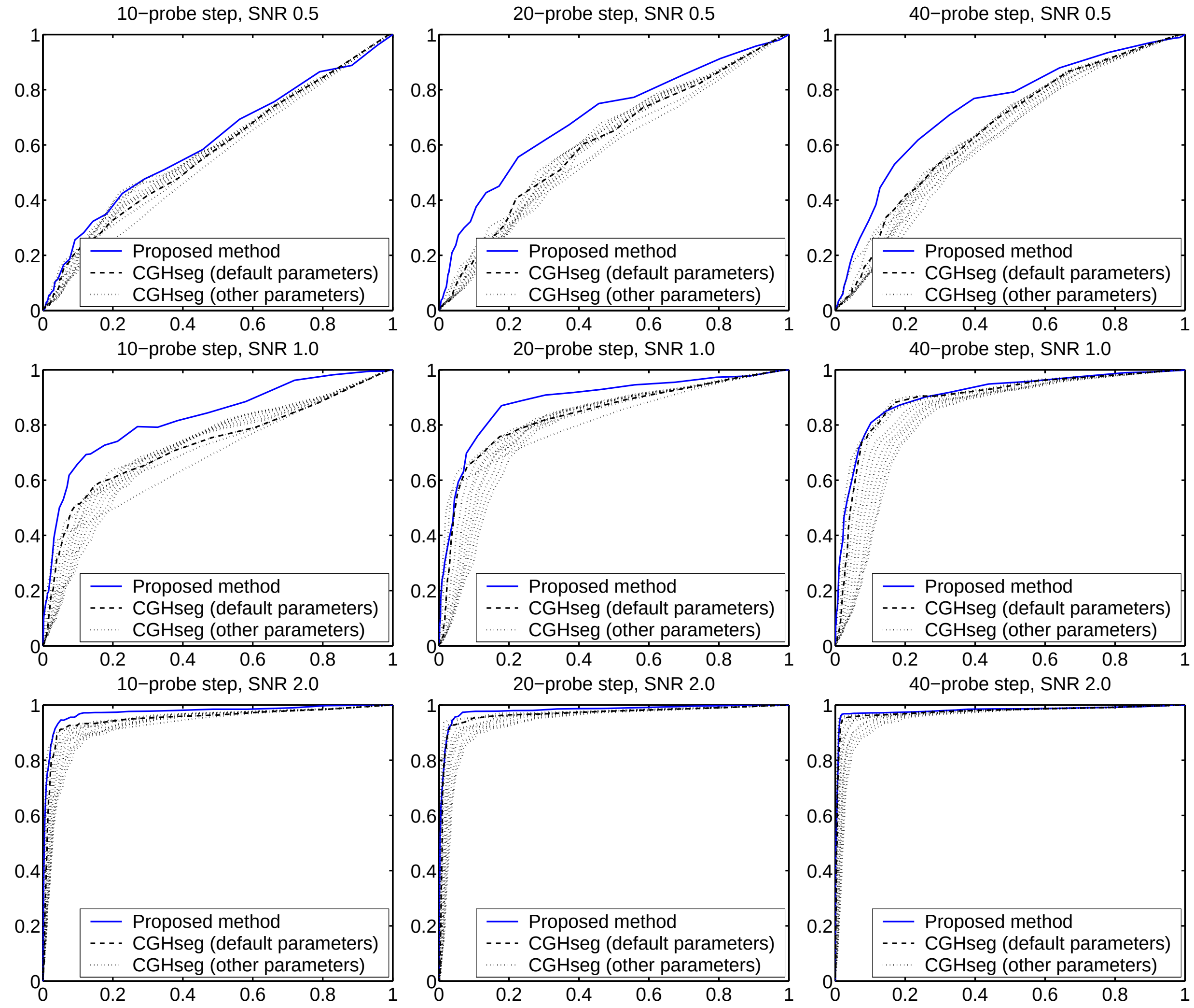

Figure 1: Effect of changing the parameter $K_{\max}$ on the receiver operating characteristics of CGH-seg. In this example, we run CGHseg with $K_{\max} = 5, 10, 20, \ldots, 100$. For values outside this range, CGHseg performed less well (data not shown). The parameter s was -0.5 (default) and the fitting model homoscedastic (default). The proportion of non-influenced genes (π) was 0.1. The other π values used in the simulation study yielded results in broad agreement with those shown.

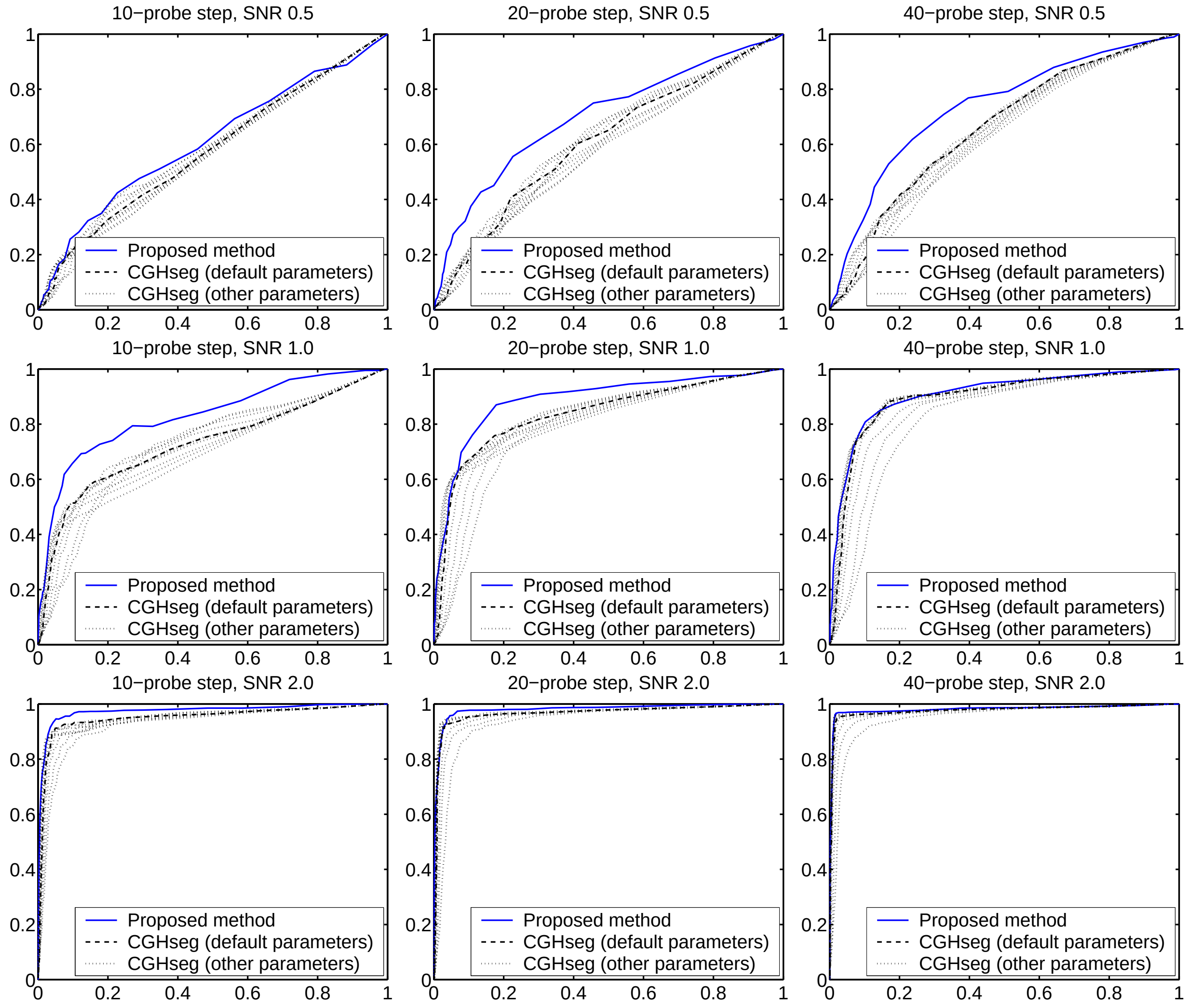

Figure 2: Effect of changing the parameter s on the receiver operating characteristics of CGHseg. In this example, we run CGHseg with $s = 0.0, -0.1, \ldots, -1.0$. For values outside this range, we obtained worse results than those shown. The parameter $K_{\max}$ was 20 (default) and the fitting model homoscedastic (default). The proportion of non-influenced genes (π) was 0.1. The other π values used in the simulation study yielded results in broad agreement with those shown.
